# High Fear of Cancer Recurrence in Chinese Newly Diagnosed Cancer Patients

**DOI:** 10.3389/fpsyg.2020.01287

**Published:** 2020-06-09

**Authors:** Xian Luo, Wengao Li, Yuan Yang, Gerald Humphris, Lijuan Zeng, Zijun Zhang, Samradhvi Garg, Bin Zhang, Hengwen Sun

**Affiliations:** ^1^Department of Psychiatry, Southern Medical University Nanfang Hospital, Guangzhou, China; ^2^School of Medicine, University of St Andrews, St Andrews, United Kingdom; ^3^Edinburgh Cancer Centre, Western General Hospital, Edinburgh, United Kingdom; ^4^Department of Organ Transplantation, Second Affiliated Hospital of Guangzhou Medical University, Guangzhou, China; ^5^CNSST Foundation New Zealand, Panmure, Auckland, New Zealand; ^6^School of Health in Social Science, University of Edinburgh, Edinburgh, United Kingdom; ^7^Department of Radiotherapy, Cancer Center, Guangdong Provincial People’s Hospital, Guangdong Academy of Medical Sciences, Guangzhou, China

**Keywords:** cancer, Chinese, fear of recurrence, newly diagnosed, structural equation modeling

## Abstract

**Background:**

Fear of cancer recurrence (FCR) is common among cancer patients and of high clinical relevance. This study explores the prevalence and correlates of FCR in Chinese newly diagnosed cancer population.

**Methods:**

This is a multicentre, cross-sectional study that includes 996 patients with mixed cancer diagnosis. All recently diagnosed patients completed a questionnaire consisting of the following: Fear of Progression Questionnaire-Short Form (FoP-Q-SF), General Anxiety Disorder Questionnaire (GAD-7), and Patient Health Questionnaire (PHQ-9). Univariate analyses, multivariate logistic regression analyses, and structural equation modeling (SEM) was performed to examine the association between tested variables and FCR.

**Results:**

Of the 996 patients, 643 (64.6%) reported high FCR (scored ≥ 34 in the FoP-Q-SF). Chemotherapy (*OR* = 1.941), Childhood severe illness experience (*OR* = 2.802), depressive (*OR* = 1.153), and anxiety (*OR* = 1.249) symptoms were positively associated with high FCR, while higher monthly income (*OR* = 0.592) was negatively associated with high FCR. SEM indicated that emotional disturbances (anxiety and depression) directly influenced FCR, while emotional disturbances partly mediated the association between personal monthly income and FCR.

**Conclusion:**

High FCR is a frequently reported problem among newly diagnosed cancer patients. Various factors increased the likelihood of the development of FCR. Flexible psychological interventions are needed for patients with high FCR.

## Introduction

Cancer is one of the important public health problems worldwide. Psychosocial concerns such as anxiety, depression, and fear of cancer recurrence (FCR) are commonly seen in cancer patients from diagnosis through survivorship (Zhao et al., 2014). FCR is often defined as: fear, worry, or concern relating to the possibility that cancer will come back or progress (Lebel et al., 2016). FCR is a key unmet need among cancer patients (Simard et al., 2013). It includes illness-related concerns, such as fear of tumor recurrence or of ongoing functional decline with all its biopsychosocial consequences (Goebel and Mehdorn, 2019). It is suggested that FCR is a unique, independent, and multidimensional construct distinct from anxiety, depression, and distress, with its own profile and mechanisms (Simard et al., 2010). Thus, it needs to be evaluated with specific instruments and treated with specific targeted psychological interventions (Simard and Savard, 2009; Thewes et al., 2012; Tauber et al., 2019).

Previous studies indicated that around 24–40% of cancer patients reported moderate to high levels of need for help dealing with FCR (Hartl, 2003; Hodgkinson et al., 2007a, b). The determinants and consequences of FCR have been preliminarily addressed by several studies. A systematic review in 2013 reported that those who diagnosed at a young age, female, and with higher education background were more likely to experience FCR compared with their counterparts (Simard et al., 2013). A new review by Fardell et al. reported that previous losses (Zakowski et al., 1997), concurrent stressors (Mellon et al., 2007), and uncertainty due to lack of information (Mishel et al., 2005) maybe important additional variables to understand the development and maintenance of clinically significant recurrence fear (Fardell et al., 2016). In addition, recent meta-analyses have revealed that mastectomy (Koch et al., 2013), radiotherapy (Yang et al., 2016), and chemotherapy (Yang et al., 2017) were significant predictors of higher FCR. Increased levels of FCR could be dysfunctional and significantly influence an individual’s well-being. It often coexists with high psychological distress and poor Health-related Quality of Life (HRQoL). Patients with elevated FCR are more likely to report anxiety, depressive, and insomnia symptoms, and report more difficulties planning for the future. FCR also negatively affects treatment adherence and may lead to higher risk of mortality (DiMatteo et al., 2000; Greer et al., 2008; Pinquart and Duberstein, 2010).

Psychological distress, such as FCR, is considered as the sixth vital sign of a cancer patient’s well-being along with signs of respiration, temperature, blood pressure, heart rate, and pain (Bultz and Carlson, 2006). However, there are only limited studies conducted in Chinese population using validated FCR measurements. Sun et al. (2019) found that around 36% of the Chinese adolescent and young adult patients experienced dysfunctional level of FCR and Yang et al. (2018) reported that life stress, childhood severe illness experience, anxiety, depressive symptom, and passive personality were independently predictive of higher FCR.

For newly diagnosed cancer patients, the days and weeks after diagnosis could be overwhelming, scary and lonely. However, inconclusive evidence was found of the association between FCR and time since diagnosis (Simard et al., 2013). There is a paucity of evidence regarding the unmet needs of newly diagnosed cancer patients. These patients have unique biological and psychological needs, therefore, more efforts should be made to provide better insights into this population.

**FIGURE 1:**
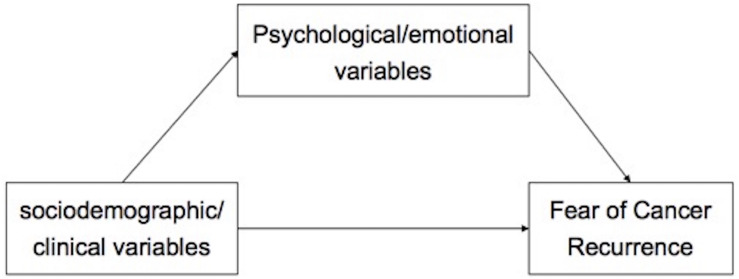
Proposed mediation path model.

In the current study, we concentrated on Chinese newly diagnosed cancer patients (≤6 months). Our aim was to assess the prevalence and correlates of high FCR. Based on previous theoretical framework and empirical evidence, the association between sociodemographic/clinical variables, and FCR were often conflictive. However, psychological/emotional variables (such as stress, anxiety, and depression) were consistently found to be risk factors of clinical levels of FCR (Chaturvedi et al., 1996; Humphris et al., 2003; Llewellyn et al., 2008; Thewes et al., 2013), and explained most of the variance in FCR (Urbaniec et al., 2011; Yang et al., 2018). Researchers suggested that anxiety and depression are causes of FCR rather than consequences (Walburg et al., 2019). Moreover, several sociodemographic/clinical variables, such as gender, financial status, and prior depression history have been proved to be risk factors for emotional disturbances (Yi and Syrjala, 2017). Therefore, we hypothesized that: (1) psychological variables (anxiety and depression) will be significantly associated with high FCR; (2) psychological variables will mediate the association between sociodemographic/clinical variable and FCR; and (3) compared to sociodemographic/clinical variables, psychological variables will have greater total effect on FCR (proposed model in Figure 1).

## Materials and Methods

### Setting and Participants

A cross-sectional study was conducted at three hospitals in southern China from September 2018 to September 2019. Patients were recruited consecutively from Department of Oncology in Southern Medical University Nanfang Hospital, Guangdong Provincial People’s Hospital, and Guangzhou Women and Children’s Medical Centre. The criteria for inclusion were: (1) age ≥ 18 years, (2) have a diagnosis of cancer within 6 months, (3) be able to read and write Mandarin or Cantonese; (4) provide written informed consent. Participants were excluded if they had severe physical and/or cognitive impairment.

### Study Procedure

All potential participants who met all eligibility criteria were asked whether they would agree to participate in the study. They were approached in the waiting room by trained research nurses who explained the study aim and procedure. Those who agreed to participate were asked to read a personal information sheet then requested to sign the written informed consent. On completion of the consenting process the participant was given a set of standardized questionnaires, and returned to the research staff immediately. This study was performed in accordance with the Helsinki standard and approved by the local ethics committee [Ref Nos: NFEC-2018-038 and 2018295H(R1)].

### Instruments

#### Demographic and Clinical Characteristics

Demographic and clinical characteristics were collected by a special-designed demographic details sheet. The collected data included: current age, gender, marital status, education, employment, personal income, type of cancer, primary treatment (surgery, chemotherapy, and radiation treatment), family cancer history, medical comorbidities, and childhood experience. Childhood adversity experience and severe illness experience were assessed by two Yes/No questions (Sun et al., 2019): (1) Have you ever experienced any childhood adversity experience, such as sexual abuse, bullying, traffic accident, or natural calamities? (2) Have you ever experienced any childhood severe illness, such as childhood cancer, or traumatic injury?

#### Fear of Cancer Recurrence (FCR)

Patient’s FCR were assessed by the 12-item Fear of Progression Questionnaire (FoP-Q-SF) (Herschbach et al., 2005). This scale has been successfully applied to samples of different cancer patients (Mehnert et al., 2006, 2009; Melchior et al., 2013). The item scores from 1 to 5 (never to very often), and the total score ranges from 12 to 60. A score of 34 or above indicates a dysfunctional level of recurrence fear (Herschbach et al., 2010). The psychometric properties of the Chinese version of FoP-Q-SF are satisfactory (Cronbach’s alpha = 0.883), and the recommended cut-off value is 34 for Chinese cancer patients (Wu et al., 2015).

#### Patient Health Questionnaire-9 Item (PHQ-9)

Patient’s depressive symptoms were assessed by the PHQ. It is a 9-item self-report measure and is commonly used in medical settings. Its items range from 0 to 3 (“not at all” to “nearly everyday”), and a total score of 5 or more indicates depressive symptoms (Kroenke et al., 2010). The Chinese version of PHQ-9 shows good psychometric properties, the Cronbach’s alpha is 0.89 (Chen et al., 2015).

#### General Anxiety Disorder Questionnaire-7 Item (GAD-7)

The GAD is a 7-item self-report scale used to measure patient’s anxiety symptoms. Response options are not at all, several days, more than half the days, and nearly every day, rated from 0 to 3, and a total score of 5 or more indicates anxiety symptoms (Spitzer et al., 2006). The GAD-7 has been translated and well-validated in Chinese language (internal consistency = 0.91) (Zheng, 2013).

### Statistical Analyses

Statistical analyses were performed using SPSS version 24.0 and Amos version 21.0. First, normal distribution assumptions were examined by one-sample Kolmogorov-Smirnov test. Descriptive statistics were used to describe all demographic and clinical characteristics of the study sample and were summarized as mean with standard deviation (SD), or frequency with percentage. Variables between the two groups (low vs. high FCR) were investigated using independent sample *t*-test, Chi-square test or Mann-Whitney *U* test, as appropriate. Second, all variables that were significant (alpha level = 0.05) in the univariate analyses were further examined by multivariate logistic regression with “enter” method. High FCR (low FCR was the reference category) was the dependent variable, while those with significant group differences in the above univariate analyses were entered as independent variables.

Finally, independent predictors of high FCR in multivariate logistic regression were further investigated by structural equation modeling (SEM), employing maximum likelihood parameter estimation, to examine the direct and indirect association between tested variables and FCR. Within the models, PHQ, GAD, and FCR variable was entered as continuous variable, and a latent variable (a hidden unobserved variable) of “emotional disturbances” was constructed to reflect the level of depressive and anxiety symptoms (Zhang et al., 2020). Spearman correlation analyses were conducted. Three different pathways were tested: (1) the path from sociodemographic/clinical variables to FCR; (2) the path form psychological/emotional variables to FCR, and (3) the path from sociodemographic/clinical variables to FCR mediated by psychological/emotional variables (Figure 1). The χ^2^/df, comparative fit index (CFI), normed-fit index (NFI), incremental fit index (IFI), Tucker-Lewis index (TLI), and Root Mean Square Error of Approximation (RMSEA) were considered as model fit indices. A CFI, NFI, IFI, and TLI of higher than 0.90, RMSEA of lower than 0.08 were indicative of good model fit (Hu and Bentler, 1999). The *P*-values less than 0.05 (two-tailed) were considered significant.

## Results

### Descriptive Statistics

A total of 1,219 patients were eligible and invited to participate. Nine hundred and ninety-six (81.71%) cancer patients agreed therefore were included in the current study. The age of participants ranged from 20 to 90 years, with a mean age of 48.04 (*SD* = 11.71) years. Approximately 90% of the participants were female, and most of them were married (85.5%), and diagnosed with breast cancer (80.6%). Of the 996 patients, 353 (35.4%) reported low FCR while the remaining 643 (64.6%) reported high FCR. Additionally, 369 (37.0%) of the patients reported depressive symptoms and 285 (28.6%) of them reported anxiety symptoms. Patient’s basic characteristics are presented in Table 1.

**TABLE 1:** Factors associated with fear of cancer recurrence (*N* = 996).

Variable	Fear of cancer recurrence					

	Total (%)	Low (%)	High (%)	X^2^	*df*	*P*
**Age**						
≤39 years	246 (24.7)	75 (21.2)	171 (26.6)			
40–60 years	556 (55.8)	221 (62.6)	335 (52.1)			
≥ 60 years	194 (19.5)	57 (16.1)	137 (21.3)	10.259	2	**0.006**
**Gender**						
Male	98 (9.8)	35 (9.9)	63 (9.8)			
Female	898 (90.2)	318 (90.1)	580 (90.2)	0.004	1	0.953
**Cancer Type**						
Breast	803 (80.6)	291 (82.4)	512 (79.6)			
Lung	109 (10.9)	39 (11.0)	70 (10.9)			
Colorectal	84 (8.4)	23 (6.5)	61 (9.5)	2.614	2	0.271
**Cancer Stage**						
Stage 1	67 (6.7)	24 (6.8)	43 (6.7)			
Stage 2	345 (34.6)	113 (32.0)	232 (36.1)			
Stage 3	507 (50.9)	187 (53.0)	320 (49.8)			
Stage 4	77 (7.7)	29 (8.2)	48 (7.5)	1.720	3	0.632
**Marital Status**						
Single	72 (7.2)	18 (5.1)	54 (8.4)			
Married	852 (85.5)	301 (85.3)	551 (85.7)			
Divorced	39 (3.9)	19 (5.4)	20 (3.1)			
Widowed	33 (3.3)	15 (4.2)	18 (2.8)	7.886	3	**0.048**
**Education Level**						
High school or below	655 (65.8)	227 (64.3)	428 (66.6)			
Undergraduate	261 (26.2)	93 (26.3)	168 (26.1)			
Postgraduate or above	80 (8.0)	33 (9.3)	47 (7.3)	1.360	2	0.507
**Personal Monthly Income**						
Less than 3,000 RMB	461 (46.3)	135 (38.2)	326 (50.7)			
3,000–5,000 RMB	258 (25.9)	98 (27.8)	160 (24.9)			
5,001–10,000 RMB	196 (19.7)	86 (24.4)	110 (17.1)			
More than 10,000 RMB	81 (8.1)	34 (9.6)	47 (7.3)	15.976	3	**0.001**
**Surgery**						
No	88 (8.8)	36 (10.2)	52 (8.1)			
Yes	908 (91.2)	317 (89.8)	591 (91.9)	1.261	1	0.261
**Chemotherapy**						
No	116 (11.6)	54 (15.3)	62 (9.6)			
Yes	880 (88.4)	299 (84.7)	581 (90.4)	7.083	1	**0.008**
**Radiotherapy**						
No	124 (12.4)	51 (14.4)	73 (11.4)			
Yes	872 (87.6)	302 (85.6)	570 (88.6)	2.002	1	0.157
**Physical Comorbidity**						
No	338 (33.9)	112 (31.7)	226 (35.1)			
Yes	658 (66.1)	241 (68.3)	417 (64.9)	1.189	1	0.276
**Family Cancer History**						
No	742 (74.5)	271 (76.8)	471 (73.3)			
Yes	254 (25.5)	82 (23.2)	172 (26.7)	1.486	1	0.233
**Childhood Adversity Exp**						
No	952 (95.6)	341 (96.6)	611 (95.0)			
Yes	44 (4.4)	12 (3.4)	32 (5.0)	1.343	1	0.247
**Childhood Severe Illness Exp**						
No	945 (94.9)	343 (97.2)	602 (93.6)			
Yes	51 (5.1)	10 (2.8)	41 (6.4)	5.890	1	**0.015**

	**M (*SD*)**	**M (*SD*)**	**M (*SD*)**	**Z**	***df***	***P***

**Depression (PHQ score)**	5.082 (4.878)	2.518 (2.933)	6.489 (5.153)	−13.721^a^	–	**<0.001**
**Anxiety (GAD score)**	3.783 (4.263)	1.510 (2.420)	5.031 (4.533)	−13.867^a^	–	**<0.001**

*In bold: *P* < 0.05; a, Mann-Whitney Test; Exp, Experience; PHQ, Patient Health Questionnaire; GAD, Generalized Anxiety Disorder 7-item scale; FCR, Fear of Cancer Recurrence.*

### Univariate Analyses

Univariate analyses revealed that high FCR was significantly associated with patient’s age (*P* = 0.006), marital status (*P* = 0.048), personal monthly income (*P* = 0.001), chemotherapy (*P* = 0.008), Childhood severe illness experience (*P* = 0.015), depressive (*P* < 0.001), and anxiety symptoms (*P* < 0.001). Patients who were younger, single, received chemotherapy, had depressive and anxiety symptoms tended to report high FCR in comparison with their counterparts. In terms of monthly income, patients with higher income were less likely to report high FCR (Table 1).

### Multivariate Logistic Regression Analyses

Multivariate logistic regression analyses showed that the receipt of chemotherapy (*OR* = 1.941, *P* = 0.008), childhood severe illness experience (*OR* = 2.802, *P* = 0.009), depressive (*OR* = 1.153, *P* < 0.001) and anxiety symptoms (*OR* = 1.249, *P* < 0.001) were positively associated with high FCR, while higher monthly income (more than 10,000 RMB, *OR* = 0.592, *P* = 0.023) was negative associated with high FCR (Table 2).

**TABLE 2:** Multivariate logistic regression of factors associated with high fear of cancer recurrence.

Variable	Category	Exp (B)	95%CI lower	95%CI upper	*P*
Age	≤39 years	Ref	–	–	–
	40–60 years	0.867	0.592	1.269	0.462
	≥60 yeaers	1.022	0.608	1.718	0.935
Gender (female)		1.292	0.756	2.207	0.348
Marital status	Single	Ref			
	Married	0.722	0.374	1.393	0.331
	Divorced	0.606	0.235	1.566	0.301
	Widowed	0.465	0.160	1.353	0.160
Personal monthly income	Less than 3,000 RMB	Ref	–	–	–
	3,000–5,000 RMB	0.768	0.525	1.125	0.175
	5,000–10,000 RMB	0.819	0.453	1.481	0.509
	More than 10,000 RMB	0.592	0.377	0.931	**0.023**
Education level	High school	Ref	–	–	–
	Undergraduate	1.234	0.832	1.830	0.297
	Postgraduate	0.860	0.488	1.515	0.601
Chemotherapy		1.941	1.189	3.170	**0.008**
Childhood severe illness exp		2.802	1.287	6.101	**0.009**
Depression (PHQ score)		1.153	1.081	1.230	**<0.001**
Anxiety (GAD score)		1.249	1.155	1.351	**<0.001**

*CI, Confidence Interval; in bold: *P* < 0.05; Exp, Experience; PHQ, Patient Health Questionnaire; GAD, Generalized Anxiety Disorder 7-item scale; FCR, Fear of Cancer Recurrence; Ref, Reference Category.*

### Structural Equation Modeling (SEM)

The results of spearman correlation analyses are shown in Table 3. Monthly income was negatively associated with depression (*r* = -0.170), anxiety (*r* = -0.125) and FCR total score (*r* = -0.136, *P* all *P* < 0.001). Chemotherapy and childhood severe illness experience showed no significant association with depression and anxiety score, and only weak association with FCR total score, which indicated that these two variables were not satisfactory to perform SEM (Yan et al., 2019; Zhang et al., 2020). Therefore, in the current study, only model of monthly income on FCR by emotional disturbances (anxiety and depression) was conducted.

**TABLE 3:** Key variables and Spearman correlation coefficients.

	1	2	3	4	5	6
1. Monthly income	1					
2. Chemotherapy	−0.181***	1				
3. Childhood severe illness exp	0.020	–0.001	1			
4. PHQ total score	−0.170***	0.024	0.024	1		
5. GAD total score	−0.125***	–0.002	0.013	0.773***	1	
6. FCR total score	−0.136***	0.093**	0.066*	0.517***	0.544***	1

*P < 0.05, **P < 0.01, ***P < 0.001. PHQ, Patient Health Questionnaire; GAD, Generalized Anxiety Disorder 7-item scale; FCR, Fear of Cancer Recurrence.

**FIGURE 2:**
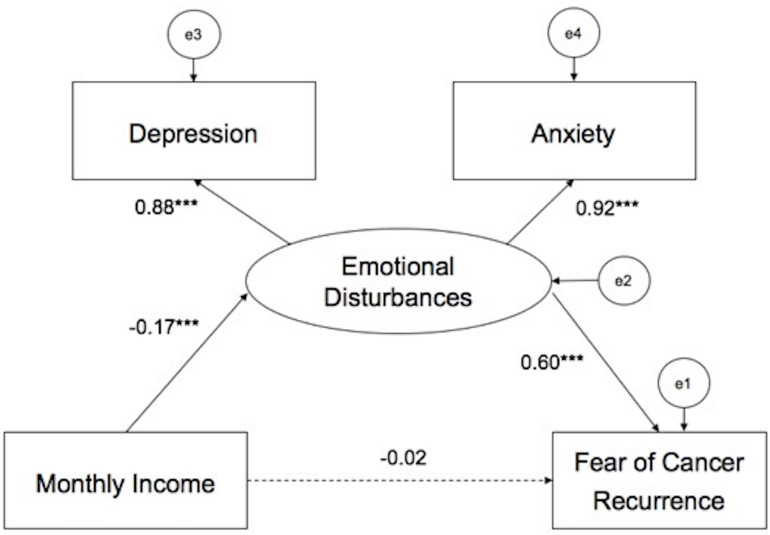
Model of monthly income on FCR by emotional disturbances. ***P < 0.001.

Figure 2 presents the model of monthly income on FCR by emotional disturbances (anxiety and depressive symptoms). Results of SEM indicated that the model had a decent fit (χ^2^/df = 7.979, CFI = 0.981, NFI = 0.979, IFI = 0.981, TLI = 0.953, RMSEA = 0.080) after controlling for age. Emotional disturbances directly influenced FCR. The standardized total effect of emotional disturbances on FCR was 0.596 (*P* < 0.05). Monthly income did not directly influence FCR, however, emotional disturbances partly mediated the association between personal monthly income and FCR. The standardized total effect of monthly income on FCR was -0.122 (standardized direct effect = -0.021, *P* > 0.05; standardized indirect effect = −0.101, *P* < 0.05).

## Discussion

In the current study, nearly 65% of the newly diagnosed cancer patients reported high FCR. One longitudinal study in 2013 reported that the prevalence rate of clinically significant FCR ranged from 44 to 56% during the cancer care trajectory, with the highest levels at a per-operative baseline, but persisting over time (Savard and Ivers, 2013). A recent study using the same assessment tool revealed that only 17% of the cancer patients reported high FCR (Gotze et al., 2019), and a similar study among young Chinese cancer population indicated that the prevalence of high FCR was around 36% (Sun et al., 2019). Cancer diagnosis and treatment is a key period of transition with important physical as well as emotional changes. For some patients, cancer diagnosis is even considered as a traumatic event capable of producing symptoms consistent with Post-traumatic stress disorder (PTSD) (Simonelli et al., 2017). The distressing symptoms experienced by newly diagnosed patients are often underreported and inadequately addressed by treating oncologists (Ghoshal et al., 2016). More supportive interventions are needed to improve the health care and quality of life for those patients.

Consistent with our first hypothesis, our study showed that patients with depression and anxiety had a higher probability to experience high levels of FCR. Literature supports the finding that there is a moderate positive association between FCR and generalized anxiety, hypochondriasis, depression, symptom distress, and psychological functioning (Chaturvedi et al., 1996; Humphris et al., 2003; Llewellyn et al., 2008; Thewes et al., 2013). Patients who experience FCR often report the chaining characteristics of worry. Those patients are more likely to spend time thinking about their disease and the risk of recurrence or progression, and they tend to have poorer psychosocial adjustment (Pedersen et al., 2012). This pattern may impair appraisal of coping responses and contribute to maintenance of fear (Simonelli et al., 2017).

The second hypothesis of the current study was partly confirmed by our study results. The association between personal monthly income and FCR was significantly mediated by emotional disturbances. Several previous findings indicated that those who were unemployed (without stable income) and those who were under great financial burdens were more likely to experience increased FCR (Skaali et al., 2009; Yang et al., 2018). However, contradictory results were also reported (Simard et al., 2013). Additionally, it has also been reported that depressive symptoms could arise as a common consequence of stress, and financial burden is considered as one important stressful live event (Halada et al., 2019). This could be a potential reason to explain the interaction between income and FCR.

Consistent with previous meta-analysis (Yang et al., 2017), chemotherapy was found to have a significant association with FCR. Patients who had been through chemotherapy may experience various side effects such as, nausea and vomiting, loss of hair, fatigue, endocrine dysfunction, and infertility (Yang et al., 2017). It has been reported that those symptoms serve as a reminder of the cancer therefore can significantly contribute to higher levels of FCR (Mehnert et al., 2009). Previous studies also showed that patients with chemotherapy are at higher risk of experience emotional distress, such as, anxiety, depression and insomnia symptoms (NHS, 2020). However, the current study found no significant association between chemotherapy and emotional disturbances. One possible reason is that more recently, patients consider chemotherapy as a neutral routine treatment and have made substantial preparation to cope with it. Health professionals also are improving their provision of information and psychosocial support for those in need.

Similarly, childhood severe illness experience was significantly associated with FCR. Evidence was found that patients with previous illness history are more likely to experience uncertainty about their future, PTSD and maladaptation (Dolgin et al., 1999). Therefore, they are at higher risk of developing FCR (Yang et al., 2018). However, the link between past illness experience and FCR should be interpreted with caution as only very few studies investigated this association in detail.

Our study findings confirmed our third hypothesis that compared to sociodemographic/clinical variables, psychological/emotional variables had greater total effect on FCR. One previous study indicated that sociodemographic and clinical variables only accounted for 7.2% of the variance in FCR, while the addition of psychological factors (anxiety, depression, and personality) increased the explained variance by 32.8% (Yang et al., 2018). Another study reported that anxiety, post-traumatic stress and functional and emotional well-being accounted for over 40% of variance in FCR (Urbaniec et al., 2011). Influential theoretical models of FCR, such as Leventhal’s Common Sense Model (CSM) (Leventhal et al., 1992; Lee-Jones et al., 1997) and cognitive theories of worry (Dugas et al., 1998), suggested that FCR shares similarities to anxiety and depressive symptoms, as patients frequently report low tolerance for uncertainty and limited future planning.

The present study has several limitations. First, several important variables, such as perceived family and social support were not investigated. Further studies are needed to explore the influence of these factors on FCR. Second, this was a cross-sectional study which could not draw any causal association between tested variables and FCR. Longitudinal studies are needed to further identify the causal association and monitor the trajectory of FCR. Third, all results were based on self-reported data, and thus recall bias may exist. Finally, some of the tested variables have floor effects, such as, the majority of the participants were females, married, and had received chemotherapy. The maldistribution of sample might cause bias, therefore, the findings cannot be generalized to the entire Chinese cancer population. Further studies with more representative samples using validated objective instruments are warranted.

This study provides several points of interest. Our study showed that high FCR is a frequently reported problem among newly diagnosed cancer population. Patient’s monthly income, the receipt of chemotherapy, childhood severe illness experience, and emotional disturbances (anxiety and depression) significantly influenced FCR. Emotional disturbances partly mediated the association between personal monthly income and FCR. These results imply that patients with these characteristics should be particularly monitored and timely psychological support should be provided when necessary.

## Data Availability Statement

The raw data supporting the conclusions of this article will be made available by the authors, without undue reservation, to any qualified researcher.

## Ethics Statement

The local Hospital Research Ethics Committee examined and approved the study [Ref Nos: NFEC-2018-038 and 2018295H(R1)].

## Author Contributions

BZ and HS: study design. XL, WL, YY, LZ, and ZZ: data collection, analysis, and interpretation. XL, WL, and YY: drafting of the manuscript. BZ, HS, GH, and SG: critical revision of the manuscript. All authors approved the final version for publication.

## Conflict of Interest

The authors declare that the research was conducted in the absence of any commercial or financial relationships that could be construed as a potential conflict of interest.
